# Probable or Possible Poisoning from Dentist’s Amalgam

**Published:** 1886-04

**Authors:** 


### THE DENTAL REGISTER.

VOL. XL.] APRIL, 1886. [No. 4.

### COMMUNICATIONS.

### Probable or Possible Poisoning From Dentists' Amalgams.

BY A PHYSICIAN.

Read before the Mississippi Valley Society.

Sometimes the deluded victims prefer to be poisoned. Sometimes they dread the dire effects of the deleterious article, yet seem to be led on, as if by siren songs, to fatal results. At other times they seem indifferent, possibly through ignorance, but oftener because the injurious results are slow in progress, and not violent in their manifestations. Even when the bad influences of the poisonous agents are liable to be transmitted from generation to generation the indifference remains. Posterity poisoned? Well, who cares? What has posterity done for us?

Still another explanation may be illustrated, and perhaps enforced by an anecdote: In the early days of our Western settlements, a preacher arrived who had lost his credentials through one of the many accidents of travel. The new parish, cautious against imposition, and believing that if really called to the ministry he would be able to preach acceptably by the aid of the Spirit, without preparatory study, proposed to test him by having the official members assign him a text as he went into the pulpit. They gave him the words, "These that have turned the world upside down are come hither also." He considered the text under four divisions: 1st, When the Creator made the world he placed it right side up. 2nd, Sin and Satan overturned it. 3d, It has been so long overturned that people regard the present as its normal position. 4th, The aim of the gospel is to turn it back to its proper state.

Now the reader can understand that if a people and nation have been constantly and continuously poisoned for generations, they will learn to regard the effects of the poison as normal manifestations of life. For want of proper ventilation all are more or less poisoned by carbonic acid, and other exhalations from living bodies. Many are poisoned from tobacco, and the symptoms are not recognized as the legitimate effects of the drug, inasmuch as as they are mild and common. Alcoholic poisoning, whether acute or chronic, though quite familiar, is often misunderstood, and the victims almost always ascribe the phenomena to other causes. Metallic poisoning is so insidious, and so gradual in its effects that the victims are usually incredulous till ruined constitutions result. The printer ascribes his ills to confinement within doors; and if his peculiar pallor is referred to, he suggests that the ink may account for it. And so it goes; and he who sounds an alarm for the purpose of arresting the evils is regarded as a crank or a fanatic.

But to discuss all the varieties of popular poisoning would take too much time and space, and as this is intended for a dental periodical, I shall only inquire as to the possibilities and probabilities of mercurial poisoning from the use of amalgam cements in filling teeth. This is a hackneyed and unpopular subject; but I think it has not received the attention from physicians that its importance demands.

I suppose it is safe to assume that many hundreds of pounds of mercurial pastes are used by dentists in a single year. It is probable that more attention is given to the preparation of these pastes than formerly, and it is possible that this renders the danger from poisoning less. If any such danger exists it is widely diffused, for probably one half the adult population who still retain their natural teeth, have more or less of this material in their mouths. And this general diffusion makes the subject very important to physicians who are the recognized conservators of the health of society. And bearing this in mind I have no apology to give to dentists for taking up the subject.

It would prove tedious to notice all the symptoms of mercurial poisoning, but at the start I will notice some of its manifestations on the nervous system.

Pereira says, under the head of "NEUROSES MERCURIALIS," "various symptoms, indicating a disordered condition of the nervous system, are met with in persons who have been exposed to the baneful influence of mercury; such as wandering pains (*neuralgia mercurialis*), a tremulous condition of the muscular system (*tremor mercurialis*), sometimes accompanied with stammering, (*psellismus metallicus*), and occasionally terminating in paralysis (*paralysis mercurialis*), epilepsy or apoplexy (*apoplexia mercurialis*). To these Dieterich adds asthma (*asthma mercurialis*), of which he saw only one case, amaurosis (*amaurosis mercurialis*), and hypochondriasis (*hypochondriasis mercurialis*)."

Pereira also says, under the head of CACHEXIA,—"this condition is characterized by disorder of the digestive organs, loss of appetite, wasting, incapability of much exertion, with increased secretion from all the organs; especially from the salivary glands."

Mr. Travers, author of *Farther Inquiry Concerning Constitutional Irritation*, says mercurial cachexia is characterized "by irritable circulation, extreme pallor and emaciation, an acute and rapid hectic and an almost *invariable termination in phthisis*." (Italics mine.)

In view of the fact that phthisis, or *consumption*, is more fatal to Americans than any epidemic pestilence, is not the last sentence worthy of the most serious consideration? And as the symptoms above detailed are very prevalent in our country or at least many of these symptoms, is it not worth while to inquire if the very general wearing of mercurial compounds in the teeth is not explanatory?

Some general principles may be considered in this connection. It is doubtful if mercury or any other metal is posionous. As long as it remains in the metallic state it is probably inert. Let the metal potassium illustrate. Grant that the metal is non-poisonous, yet indirectly its introduction may result in serious disaster. It has such a strong affinity for oxygen that it will burn briskly in water; and its oxide formed thus, or otherwise, is a caustic poison of deadly power.

Mercury combines with oxygen forming two compounds, both poisonous. And all its compounds with non-metallic elements are poisonous, unless its sulphides are to be excepted. As these are but slightly soluble, some claim that they are non-poisonous or inert. But even if this is true it does not follow that they can be used with safety, for they are readily decomposed, and thus the mercury may, in part, be taken from the sulphur by its affinity for other substances, and thereby poisonous compounds may be formed.

The facts that sulphur has a strong affinity for mercury, that the sulphides of mercury are not very soluble, and that many mouths, perhaps a majority, contain sulphur in the form of sulphurated hydrogen, or cyanide of sulphur (sulpho-cyanogen), explain why we do not see more frequent and more violent poisonings from amalgam fillings than are observable. Metallic zinc is highly oxidizable, but the first resulting compound of the two elements, the sub-oxide of zinc forms an insoluble coating on the surface of the metal, which shuts out remaining oxygen, thus arresting the process of oxidation. In like manner sulphur forms, with the mercury of an amalgam filling, a protecting coat that to a good extent shuts out oxygen, chlorine, etc. Were this otherwise it is doubtful if any could wear amalgam fillings with impunity.

But still, from the standpoint of the physician, it is difficult to see, that in view of the general use of mercury in filling teeth, cases of poisoning do not frequently occur. Of course very many, and probably a great majority of the cases are so mild in their results that the real cause is overlooked. The constitution has been so long overturned that its present is regarded as its normal position, and all the more in view of the fact that the overturning at the first was accomplished by such a gradual process.

In 1850 I was called to treat a young school girl for *paralysis agetans.* She had been under treatment by other physicians without benefit, and for several weeks I could see no improvement. After a time I saw she had quite a number of amalgam fillings in her teeth, and these were promptly removed, after which she gained rapidly under treatment that had altogether failed before.

A strong man of thirty had in his mouth twelve large mercurial fillings. His physician decided that he was suffering from mercurial ptyalism. His salivary glands and his tongue were much swollen. The accompanying stench was sickening. When called for counsel I could but approve the diagnosis, and the history of the case proves its accuracy. These are but specimen cases, I have seen many others as well marked. Of course they are exceptions, for if such results were the rule the material would be promptly abandoned. Yet I have no doubt, whatever, that many of the aches and pains complained of in society arise from mild, chronic mercurial poisoning.

I suppose amalgam fillings will continue to be used; but it is well to use them with open eyes. If some are injured, and many apparently escape all trouble, and certainly do escape serious results, it becomes an important matter to find out what peculiarities of constitution make them more objectionable. If this were well understood, it could be omitted in treating such.

I think all first class scientific observers will recognize that there is some danger from this source; and if poisoning may so result, how is it that the poisoning occurs?

Dr. Talbot, of Chicago, in a paper found at page 123, vol. v. of the *Ohio Journal*, seems to think that a vaporization of the mercury is the prominent factor in this form of poisoning. Long ago Dr. Watt and others suggested chloridation of the mercury. The two views are perfectly compatible. By vaporization a greater surface of the mercury is exposed to chemical action, and therefore oxygen, and also chlorine have better opportunities to combine with it; and it is well known that both the oxides and chlorides are active poisons.

Some have objected that the vapor theory will not hold because a temperature of several hundred degrees is required to vaporize mercury, but the statement was made evidently without thought. Water *boils* at 212°, but it vaporizes at all known temperatures, and in like manner mercury does not require the boiling point to convert it into vapor. Besides, mercury has such small capacity for heat that notwithstanding its high boiling degree, it is boiled with less heat than that required for water. A heat that raises the temperature of water but *one* degree, raises mercury *thirty* degrees. This in part explains its ready evaporation.

It has been claimed that evaporation does not take place from amalgam fillings in the mouth because they weigh as much after a lapse of months as when first put in. It is natural to expect an increase of weight. Dr. Talbot, I think, claims that such increase proves porosity of the material, and is due to the absorption of water. But if chemical agents, as oxygen, sulphur, or chlorine, combine with any of the metals, there must be an increase in weight.

But in many cases the buccal fluids hold soluble chlorides in solution. They usually, at least often, divide their chlorine with other elements for which it has an affinity. In this way chloridation of mercury may take place, whether it be in vapor or otherwise. And it is well known that mercuric chloride (corrosive sublimate) is very poisonous. Galvanic action within the mouth is usually met with a sneer, when mentioned. I trust that as you made it the subject of your inaugural thesis, when trying for the degree of D.D.S., you will not be too much prejudiced to listen to something said about it. A tin filling and a gold one may be so placed as to make a battery, the tin may be thus made positive and the gold negative. But that a current may be formed, and eclectrolysis result, they must be connected by a conductor. The gums, lips, or tongue may make such connection. Still that a current may be formed, one of the metals must be corroded. In this way any of the binary compounds of the buccal fluids may be decomposed. If chlorine is thus liberated, it at once takes hydrogen from the water present to form hydrochloric acid.

But galvanic action and electrolysis may occur without such arrangement, even from a single amalgam filling. Let a fragment of commercial zinc be placed in water, and a portion of the water is at once decomposed. The surface of the metal becomes quickly quoted with an oxide, and the action ceases. By adding a minute quantity of acid to dissolve the oxide as fast as formed, the decomposition continues. Pure zinc will thus decompose water, but not so rapidly as commercial zinc, because the latter has diffused through it minute particles of iron and other metals. At the point of contact of the zinc with the iron, rapid action takes place, because of the galvanic action thus set up. And this is as truly electrolysis as anything known in science.

Now apply this to an amalgam filling in the mouth: Several metals are commingled, minute points of any metal acted on more rapidly than other metals in contact with it, give us a series of minute galvanic batteries, such one having great decomposing power.

By lessening the space between the exciting plates of a battery one half, its decomposing power is increased four fold. Now, lessen the space to an infinitessimal distance, and we can scarcely estimate the power, it becomes so great in proportion to the space so excited from such small point. In this way the metal most readily corroded by chlorine suffers more than the others.

I have seen trouble from amalgam fillings totally different from anything yet named here. A man was wearing a gutta-percha filling in a lower molar. His dentist expected to fill with gold in a few weeks or months. A traveling jack persuaded him he would lose the tooth unless immediately filled with gold. So he filled it nearly full with amalgam, and finished with gold. The patient began to suffer at once and was told that the distress would soon abate. He called on his family dentist repeatedly and was angry because he would not relieve him without removing the filling, which he insisted was all gold. After two or three weeks of almost sleepless distress, the plug was removed with instant relief. Now this is not likely a case of "intrastomatic galvanism," but the trouble was probably caused by thermo-electricity.

I have seen cases like the above when gold and tin had been used in the same cavity. But this is not common, for these metals are often used together without any inconvenience. Unless one metal is corroded no galvanic action can occur in such cases; and they bear no analogy to metallic poisoning.

Of course amalgam fillings will be used. They are too convenient to be laid aside. But I wish to suggest that physicians in diagnosing obscure cases, give more attention to this possible source of mercurial poisoning. It will not do to deny that poisoning sometimes occurs from this source. Many dentists of high repute, though using the cement, testify to seeing well-marked cases. Other dentists express doubts about such poisoning, because they have never seen cases of the kind ; and one got letters from physicians who state they had never seen cases of such poisoning. But what does negative testimony amount to in a controversy like this ? I know a dentist of thirty years practice who has never seen an amalgam filling inserted, yet he believes one has been used.

It is often maintained that the observers who claim to have seen mercurial ptyalism from this source are mistaken as to the cause. And some remind us that iodide of potassium can cause ptyalism similar to that from mercury. I believe it can do so only when mercury has been previously lodged in the system.

